# Prevalence and correlates of physical and sexual intimate partner violence among women living with HIV in Uganda

**DOI:** 10.1371/journal.pone.0202992

**Published:** 2018-08-27

**Authors:** Cynthia R. Young, Angela Kaida, Jerome Kabakyenga, Winnie Muyindike, Nicholas Musinguzi, Jeffrey N. Martin, Peter W. Hunt, David R. Bangsberg, Jessica E. Haberer, Lynn T. Matthews

**Affiliations:** 1 Division of Women’s Health, Brigham and Women's Hospital, Boston, MA, United States of America; 2 Faculty of Health Sciences, Simon Fraser University, Burnaby, BC, Canada; 3 Maternal Newborn and Child Health Institute, Mbarara University of Science & Technology, Mbarara, Uganda; 4 Department of Internal Medicine, Mbarara University of Science & Technology, Mbarara, Uganda; 5 Global Health Collaborative, Mbarara University of Science & Technology, Mbarara, Uganda; 6 Department of Epidemiology & Biostatistics, University of California San Francisco, San Francisco, CA, United States of America; 7 Department of Medicine, University of California San Francisco, San Francisco, CA, United States of America; 8 Oregon Health & Science University-Portland State University School of Public Health, Portland, OR, United States of America; 9 Center for Global Health, Massachusetts General Hospital and Harvard Medical School, Boston, MA, United States of America; The Ohio State University, UNITED STATES

## Abstract

**Background:**

Intimate partner violence (IPV) is a significant global health problem. Women who experience IPV have increased HIV incidence, reduced antiretroviral adherence, and a lower likelihood of viral load suppression. There is a lack of evidence regarding how to effectively identify and support women living with HIV (WLWH) experiencing IPV, including uncertainty whether universal or targeted screening is most appropriate for lower-resourced settings. We examined physical and sexual IPV prevalence and correlates among WLWH in Uganda to understand the burden of IPV and factors that could help identify women at risk.

**Methods:**

We utilized data from women receiving ART and enrolled in the Uganda AIDS Rural Treatment Outcomes (UARTO) cohort study between 2011 and 2015. Bloodwork and interviewer-administered questionnaires were completed every 4 months. IPV was assessed annually or with any new pregnancy. Multivariate models assessed independent socio-demographic and clinical factors correlated with IPV, at baseline and follow-up visits.

**Results:**

455 WLWH were included. Median age was 36 years, 43% were married, and median follow-up was 2.8 years. At baseline 131 women (29%) reported any experience of past or current IPV. In the adjusted models, being married was associated with a higher risk of baseline IPV (ARR 2.33, 95% CI 1.13–4.81) and follow-up IPV (ARR 2.43, 95% CI 1.33–4.45). Older age (ARR 0.96, 95% CI 0.94–0.99) and higher household asset index score (ARR 0.81, 95% CI 0.68–0.96) were associated with lower risk of IPV during follow-up.

**Conclusion:**

There was a high prevalence of physical and sexual IPV amongst WLWH, and many women experienced both types of violence. These findings suggest the need for clinic-based screening for IPV. If universal screening is not feasible, correlates of having experienced IPV can inform targeted approaches.

## Introduction

Intimate partner violence (IPV) is defined as behavior within an intimate relationship that causes physical, sexual, or psychological harm. IPV is an important component of gender inequality and a global public health problem. The lifetime prevalence of physical or sexual IPV among women worldwide is 30% [1,2]. The dual global epidemics of IPV and HIV are closely related [3]. Women living with HIV (WLWH) may be at increased risk of IPV [3], IPV is associated with HIV incidence [4,5], and IPV is associated with reduced antiretroviral (ART) adherence and a lower likelihood of viral load suppression [6,7]. These relationships have important implications for HIV transmission and treatment, and make understanding and intervening on IPV a high priority for the care of WLWH.

Estimates of IPV prevalence among women in Uganda vary. In a community cohort in Uganda, past-year prevalence of physical IPV was 18% and sexual IPV was 15% [8]. The Uganda Demographic and Health Survey found 36% of women had ever experienced physical IPV and 22% of women had ever experienced sexual IPV [9]. In a study across 10 countries, risk factors for physical or sexual IPV included younger age, alcohol abuse, childhood experience of abuse or domestic violence, and prior experience of IPV. Secondary education, high socioeconomic status, and formal marriage were protective [10].

There is broad support for integrating screening and treatment of IPV into healthcare settings and approaches to trauma-informed care have been developed for WLWH [2,11,12]. However, these care models largely come from higher-resourced healthcare settings [2,13]. The role of universal versus targeted IPV screening for women presenting to care is controversial, and targeted screening may be more appropriate for lower-resourced healthcare settings [14–17]. While protocols for sexual assault have been implemented in HIV care in Uganda, comprehensive care for women experiencing IPV is lacking [18]. To better understand the burden of IPV in women accessing HIV care, and whether there are factors that could be helpful for identifying women at risk, we examined IPV prevalence and correlates among WLWH in Uganda. Understanding the burden of IPV and factors associated with IPV in settings like Uganda, with a high IPV prevalence and a generalized HIV epidemic, is an important step for developing effective IPV screening and treatment interventions.

## Materials and methods

### Study setting

Mbarara Town (population 195,000) is located in the Mbarara District of Uganda, 275 km southwest of the capital city, Kampala. The Mbarara Regional Referral Hospital (MRRH) HIV clinic initiates ART in more than 1,000 patients per year and offers comprehensive HIV care services, including ART provided through the Ugandan Ministry of Health with support from the President’s Emergency Plan for AIDS Relief, the Global Fund, and the Family Treatment Fund [19].

### Study participants

We utilized prospective data from women enrolled in the Uganda AIDS Rural Treatment Outcomes (UARTO) cohort study, which recruited treatment-naive adults initiating ART at the MRRH HIV clinic. This study ran from 2005–2015 with the primary objective of understanding ART adherence, virologic failure, and associated factors. Clinic patients who were at least 18 years old and living within 60 km of the clinic were eligible to enroll in the study. This analysis utilizes data from the Reproductive Health Component of the study, which ran from October 2011 through September 2015. Bloodwork (CD4 cells/mm3, HIV-RNA), urine beta-hCG, and interviewer-administered questionnaires (socio-demographics, behavior, and health outcomes) were collected every 4 months. Sexual and reproductive health data, including experience of IPV, were collected annually and at the time of any new pregnancy. Women were included in this analysis if they completed the Reproductive Health Component questionnaire at least once.

### Measurements

Questions about IPV were adapted from the Conflicts Tactics Scale [20], which has been used in studies of IPV in sub-Saharan Africa [8,21,22]. A history of lifetime experience of physical or sexual IPV was assessed at baseline with the following questions:

- Have any of your sexual partners ever done any of the following: Pushed, pulled, slapped, or held you down? Punched you? Kicked you or dragged you? Tried to strangle or burn you? Threatened or attacked you with a gun/knife/other weapon?
- Has anyone ever physically forced you to have sex with him when you did not want to?

Current IPV was assessed at baseline and follow-up visits, and is defined as any report of experiencing physical and/or sexual IPV in the past 12 months, or since the form was last completed. Current experience of physical or sexual IPV was assessed with the following questions:

- In the past 12 months (or since last time you completed this form), have any of your sexual partners done any of the following: Pushed, pulled, slapped, or held you down? Punched you? Kicked you or dragged you? Tried to strangle or burn you? Threatened or attacked you with a gun/knife/other weapon?
- In the past 12 months (or since last time you completed this form), has anyone physically forced you to have sex with him when you did not want to?

Socio-demographic and clinical factors assessed including age (by year), education (no school, primary only, secondary or greater), employment status (unemployed vs. employed), marital status (married vs. not married), age at sexual debut (≤15 years vs. >15 years), social support (measured with the Social Support Scale: mean score of 10 items scored on a 4-point scale, higher scores indicate greater social support) [23,24], hazardous alcohol use (AUDIT-C score, with cutoff score of ≥3 indicating hazardous alcohol use) [25,26], and probable depression (score ≥1.75 on Hopkins Symptom Checklist) [27–29]. We also included a measure of household asset wealth index based on the methodology of Filmer and Pritchett [30], which includes data on durable goods, quality of housing, and the sources of energy available in the household.

### Data analysis

All covariates were obtained when participants first completed the Reproductive Health Component questionnaire, with the exception of age at sexual debut which was asked at study enrollment. Descriptive statistics were used to assess key characteristics of study participants. The primary outcome of interest was any report of current physical and/or sexual IPV during the study period (yes vs. no). We examined associations between covariates and current IPV using Fisher’s Exact Test for categorical variables and the Wilcoxon rank sum test for continuous variables. We fit logistic regression models to assess correlates of current IPV at the baseline study visit, and of IPV during follow-up study visits. All significant covariates from the unadjusted analyses (p<0.25) were selected for the adjusted logistic regression model [31]. Statistical tests were two-sided, and significance was determined at the alpha = 0.05 level. Data analysis was performed in Stata V14.2 (StataCorp LLC, College Station, TX).

### Ethics considerations

Ethical approval for all study procedures was obtained from the Institutional Review Committee, Mbarara University of Science and Technology; the Uganda National Council of Science and Technology; the Partners Human Research Committee, Massachusetts General Hospital; the Committee on Human Research, University of California at San Francisco; and the Research Ethics Board of Simon Fraser University. All participants provided voluntary, written informed consent at study enrollment. Women reporting experience of violence were referred to locally available counseling services.

## Results

A total of 455 women contributed to the analysis (Table 1). Of those, median age at baseline was 36 years (IQR: 29–43) and median time on ART was 4 years (IQR: 0–5.1). 196 (43%) were married, 119 (26%) were widowed, 110 (24%) were divorced/separated, and 30 (7%) were never married. Women reported a median of 1 sexual partner in the past 12 months (IQR: 0–1). Of the 316 women (69%) with at least 1 recent sexual partner, 252 (80%) reported that their partner knew their HIV status at the last sexual encounter, and 219 (69%) reported that they knew their partner’s HIV status at the last sexual encounter. Women were followed for a median of 2.8 years (IQR: 2.1–3.0), contributing 1,161 person years of follow-up.

**Table 1:** Baseline characteristics of HIV-infected women accessing ART in rural Uganda.

Variable	N	Median (IQR)	n (%)
Age (years)	454	36 (29–43)	
Household asset index score	455	-0.2 (-1.5–1.3)	
Education	455		
*No school*			87 (19%)
*Primary only*			242 (53%)
*Secondary or greater*			126 (28%)
Not employed	455		132 (29%)
Relationship status	455		
*Married*			196 (43%)
*Widowed*			119 (26%)
*Divorced/Separated*			110 (24%)
*Never married*			30 (7%)
Number of sexual partners in last 12 months	455	1 (0–1)	
Primary partner knows woman's HIV status	316*		252 (80%)
Primary partner’s HIV status	316*		
*HIV positive*			160 (51%)
*HIV negative*			59 (19%)
*Don't know*			97 (31%)
CD4 count (cells/mm^3^)	449	392 (286–536)	
HIV-1 RNA suppression (on ART ≥ 6 mo)	323		301 (93%)
Years on ART	455	4.0 (0–5.1)	
Age at sexual debut ≤15 years	442		102 (23%)
Social support scale score	455	3.2 (2.7–3.7)	
Hazardous alcohol use (AUDIT-C)	455		20 (4%)
Probable depression (HSCL-D)	455	56 (12%)	

*316 women with ≥1 sexual partner at baseline

**Fig 1:**
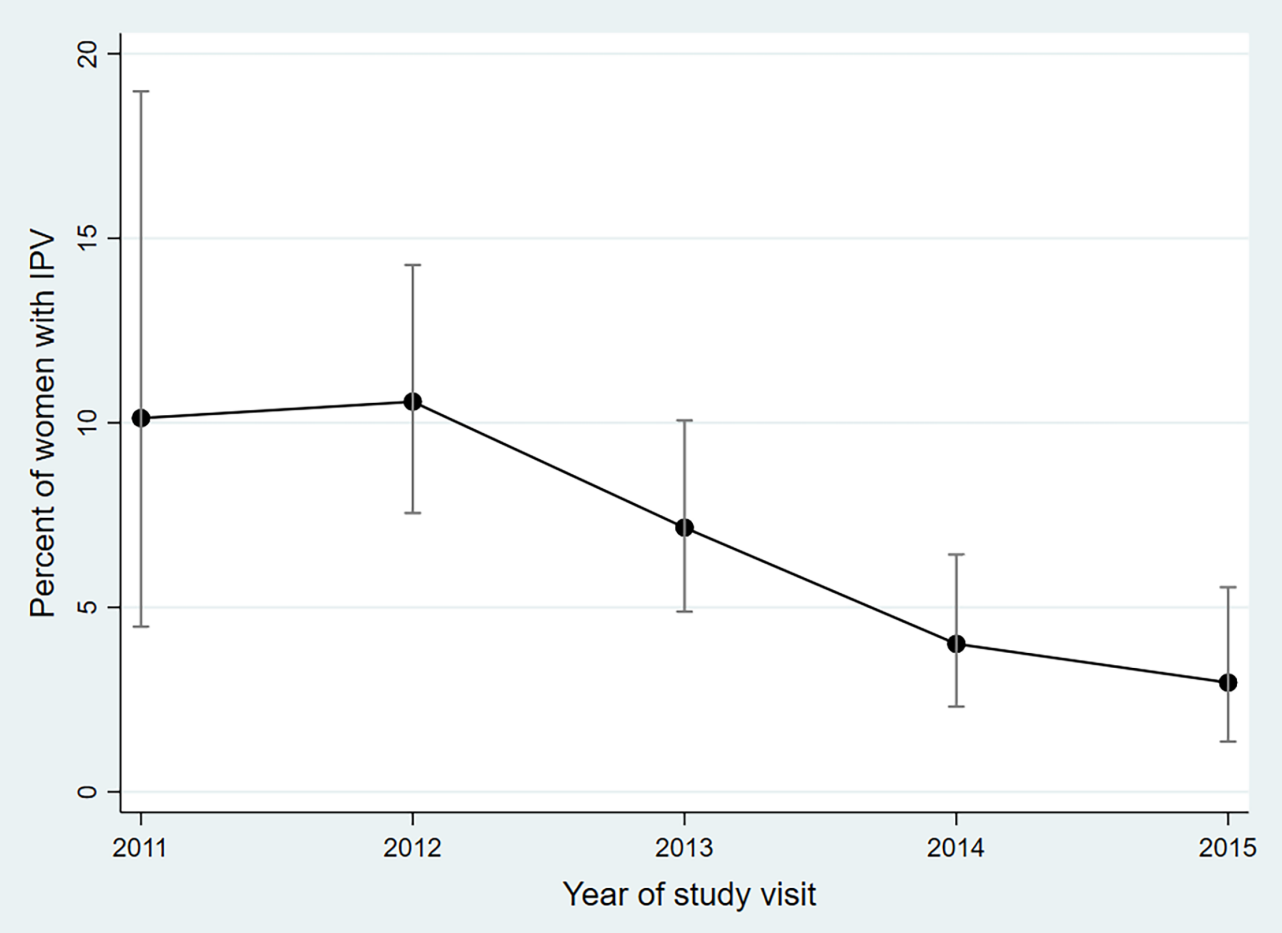
Percent of women who report current physical and/or sexual IPV, with 95% CI (N = 1,813 study visits).

At baseline, 131 women (29%) reported any experience of past or current IPV. Of those, 65 (50%) experienced physical violence only, 23 (18%) experienced sexual violence only, and 43 (33%) experienced both. An average of 6% of women per year reported current IPV, but reporting of IPV declined over time (Fig 1). Current IPV was reported by 10% of women in 2011, 11% of women in 2012, 7.2% of women in 2013, 4.0% of women in 2014, and 3.0% of women in 2015 (p<0.001, chi-squared test for trend).

34 women (7.5%) reported current IPV at their baseline visit. Of those, 9 (26%) reported physical violence only, 16 (47%) reported sexual violence only, and 9 (26%) reported both. 50 women (11%) reported current IPV at least once during study follow-up. Of those, 17 (34%) reported physical violence only, 19 (38%) reported sexual violence only, and 14 (28%) reported both.

We developed logistic regression models to assess correlates with current IPV at baseline, and with current IPV during follow-up study visits. For current IPV at baseline, 431 women contributed complete data (Table 2). Of the women excluded from the analysis, 2 were missing data on current IPV at baseline, 13 were missing data on age at sexual debut, 6 were missing employment status, and 3 were missing household asset index. Household asset index, education, age at sexual debut, and social support were excluded from the adjusted model. In the adjusted model, being married (ARR 2.33, 95% CI 1.13–4.81) was associated with higher risk of experiencing current IPV.

**Table 2:** Estimates from unadjusted and adjusted logistic regression analyses to identify factors associated with intimate partner violence at baseline (N = 431 women).

Variable	Unadjusted RR	95% CI	p-value		Adjusted RR	95% CI	p-value
Age, years	0.96	0.92–0.99	0.018		0.98	0.94–1.02	0.316
Household asset index score	0.96	0.83–1.11	0.554				
Education							
*No school*	1						
*Primary only*	1.28	0.49–3.34	0.616				
*Secondary or greater*	1.39	0.49–3.92	0.534				
Not employed	2.13	1.11–4.10	0.023		1.83	0.94–3.55	0.074
Married	2.59	1.28–5.20	0.008		2.33	1.13–4.81	0.022
Age at sexual debut ≤ 15 years	1.07	0.50–2.31	0.856				
Social support scale score	1.09	0.73–1.62	0.671				
Hazardous drinking	2.83	1.10–7.29	0.031		2.61	0.98–6.98	0.056
Probable depression	2.19	1.04–4.61	0.039		1.97	0.91–4.23	0.083

For current IPV at follow-up visits, 420 women contributed complete data (Table 3). Of the women excluded from the analysis, 13 were missing data on IPV during follow-up, 13 were missing age at sexual debut, 6 were missing employment status, and 3 were missing household asset index. Education and depression were excluded from the adjusted model. In the adjusted model, being married (ARR 2.43, 95% CI 1.33–4.45) was associated with higher risk of experiencing current IPV, while older age (ARR 0.96, 95% CI 0.94–0.99) and higher household asset index (ARR 0.81, 95% CI 0.68–0.96) were associated with lower risk of IPV.

**Table 3:** Estimates from unadjusted and adjusted logistic regression analyses to identify factors associated with intimate partner violence during study follow-up (N = 420 women).

Variable	Unadjusted RR	95% CI	p-value		Adjusted RR	95% CI	p-value
Age, years	0.95	0.93–0.98	0.001		0.96	0.94–0.99	0.011
Household asset index score	0.83	0.70–0.97	0.022		0.81	0.68–0.96	0.017
Education							
*No school*	1						
*Primary only*	1.31	0.62–2.74	0.482				
*Secondary or greater*	0.98	0.41–2.32	0.958				
Not employed	2.01	1.19–3.42	0.010		1.46	0.85–2.48	0.167
Married	2.62	1.48–4.62	0.001		2.43	1.33–4.45	0.004
Age at sexual debut ≤ 15 years	1.66	0.95–2.90	0.072		1.52	0.86–2.68	0.153
Social support scale score	0.78	0.56–1.07	0.127		0.94	0.69–1.30	0.717
Hazardous drinking	2.03	0.82–5.04	0.127		1.5	0.58–3.85	0.402
Probable depression	1.51	0.75–3.05	0.245				

## Discussion

In this cohort of women accessing HIV care in rural Uganda, 131 women (29%) reported any experience of physical or sexual IPV at baseline. An average of 6% of women per year reported current physical or sexual IPV, but reporting declined over time. At baseline, 7.5% of women reported physical or sexual violence in the past 12 months. While our data is similar to global lifetime IPV prevalence estimates, it shows a lower prevalence of IPV than other studies in Uganda [8,9]. The differences in our analysis may be due to several factors, including selection bias. The women in our cohort, who accessed ART and participated in a longitudinal cohort study, may be relatively advantaged compared to the general population. The women in our cohort were older than the general population [32], and 28% of women in our sample had some secondary education or greater, versus 16% of Ugandan women nationally [33]. 93% of women in this cohort on ART at least 6 months had HIV-1 RNA suppression, while only 63% of women in Uganda living with HIV are virally suppressed [34]. Nonetheless, our findings demonstrate that IPV is a common health problem among women accessing HIV care. The reasons for the decline in IPV reporting over time are unclear. Our study protocol included referral for women reporting IPV, though we do not know what proportion of women accepted that referral.

In the adjusted models, being married was associated with an increased risk of IPV, and younger age and lower household asset index were associated with an increased risk of IPV during study follow-up. The association between marital status and IPV is mixed in the literature. Marriage may indicate a higher likelihood or frequency of intimate partner contact. In most studies, being single, separated, or divorced is associated with increased risk of IPV [1,35]. In another community in Uganda, being in a relationship with a husband carried more risk for IPV [8,36]. Younger age and lower socio-economic status have been associated with IPV in other studies [1], and seems to be a shared risk factor across diverse settings and populations of women. It is unclear why these associations were present only for IPV during study follow-up, and merits further investigation.

Limitations of this analysis include the fact that under-reporting of IPV is possible, and we did not measure emotional IPV. The WHO multi-country survey used a longer and more detailed questionnaire to measure IPV, which may facilitate disclosure [37,38]. This was also a cross-sectional analysis so could not assess causality.

## Conclusion

This analysis shows a large burden of IPV within a population of Ugandan women accessing ART and engaged in HIV care. Within the setting of chronic HIV care with regular follow-up, we are missing opportunities to address IPV and the subsequent negative health consequences. Models of care to address IPV in women accessing healthcare have not been widely developed and tested for sub-Saharan Africa, but trauma-informed care models for WLWH in the United States may serve as a guide [12]. Our findings suggest the need for clinic-based screening for IPV in this setting. If universal screening is not feasible, correlates of IPV such as marital status, younger age, and lower socio-economic status can inform targeted approaches.

## Supporting information

S1 File Analysis dataset. (XLSX)
